# Locking Src/Abl Tyrosine Kinase Activities Regulate Cell Differentiation and Invasion of Human Cervical Cancer Cells Expressing E6/E7 Oncoproteins of High-Risk HPV

**DOI:** 10.1155/2010/530130

**Published:** 2010-08-25

**Authors:** Amber Yasmeen, Amal Alachkar, Hafedh Dekhil, Carlo Gambacorti-Passerini, Ala-Eddin Al Moustafa

**Affiliations:** ^1^Segal Cancer Centre, Lady Davis Institute for Medical Research of the Sir Mortimer B. Davis-Jewish General Hospital, Davis-Jewish General Hospital, McGill University, 3755, Ch. de la Cote Ste-Catherine, Montreal, QC, Canada H3T 1E2; ^2^Faculty of Pharmacy, University of Aleppo, Aleppo, Syria; ^3^Syrian Research Cancer Center of the Syrian Society against Cancer, Aleppo, Syria; ^4^Department of Clinical Medicine and Prevention, University of Milano-Bicocca, 20052 Monza, Italy; ^5^Department of Mechanical Engineering, Concordia University, Montreal, QC, Canada H4B 1R2; ^6^Department of Oncology, Faculty of Medicine, McGill University, QC, Canada H3G1M8

## Abstract

In this study, we compared the effects of SKI-606 with Iressa, Src/Abl and EGF-R kinase inhibitors, respectively, on selected parameters in HeLa and SiHa cervical cancer cell lines, which express E6/E7 oncoproteins of high-risk HPV types 18 and 16, respectively. Our results show that SKI-606 and Iressa inhibit cell proliferation and provoke G_0_-G_1_ cell cycle arrest and reduction of S and G_2_-M phase using 2 and 5 *μ*M concentrations of these inhibitors. In contrast, SKI-606 induces differentiation to an epithelial phenotype “mesenchymal-epithelial transition”; thus SKI-606 causes a dramatic decrease in cell motility and invasion abilities of HeLa and SiHa cancer cells, in comparison to untreated cells and Iressa-treated cells in which these parameters are only slightly affected. These changes are accompanied by a regulation of the expression patterns of E-cadherin and catenins. The molecular pathway analysis of Src/Abl inhibitor revealed that SKI-606 blocks the phosphorylation of *β*-catenin and consequently converts its role from a transcriptional regulator to a cell-cell adhesion molecule. Our findings indicate that SKI-606 inhibits signaling pathways involved in regulating tumor cell migration and invasion genes via *β*-catenin alteration, suggesting that Src inhibitor, in comparison to EGF-R, is a promising therapeutic agent for human cervical cancer.

## 1. Introduction

Cervical cancer is the second most common malignancy amongst women worldwide with approximately 500,000 new cases and 250,000 deaths each year estimated by the World Health Organization. Thus, the overall 5-year-survival rate for patients diagnosed with cervical cancer is approximately 50% and has not significantly improved over the past two decades. Important etiological factors in the development of this cancer are human papillomaviruses (HPVs), as more than 96% of cervical cancers are positive for high-risk HPVs especially types 16, 18, 31, 33, and 35 [1–3]. Moreover, accumulating evidence suggests that persistent infection with these viruses is necessary for cervical precursors to evolve into invasive carcinomas [4–7]. The E6 and E7 oncoproteins of high-risk HPVs, which are constitutively expressed in these cancers, inactivate p53 and pRb tumour suppressors, respectively [8]. E6 facilitates the degradation of p53 through its association with an accessory protein, E6-AP, a component of the ubiquitin proteolytic pathway [9]. E7 proteins of high-risk HPVs bind to Rb [10], as well as to other pocket proteins, such as p107 and p130 [11], leading to cell cycle deregulation. This results in genomic instability and has been implicated in the transformation and progression of normal and cancerous cervical cells, respectively.

Human cervical cancers are characterized by a marked propensity for invasion and spreading to local lymph nodes. Several investigations indicate that both invasion and metastasis may be critically dependent on the acquisition of the epithelial-mesenchymal transition (EMT) process [12, 13]. At least, three coordinated processes are necessary for invasion: (1) changes in cell-cell and cell-matrix adhesion, (2) degradation of the extracellular matrix (ECM), and (3) cell migration. Therefore, cell-cell adhesion proteins are greatly involved in these processes. Several studies reported that the E-cadherin/catenin complex and P-cadherin, of the cell-cell adhesion family, as well as fascin, Id-1, IGF-R1, and EGF-R genes are important regulators in the progression of several human carcinomas including cervical [14–18]. In contrast, earlier studies reported that Src activation induces phenotypic traits associated with metastatic cancer cells, such as increased cell invasiveness and motility thus decreasing the survival of numerous human cancer patients [19, 20].

To explore the role of Src/Abl inhibitor, SKI-606, as a potential treatment for human cervical carcinomas, we compared the effect of SKI-606 with Iressa on cell proliferation, cell cycle progression, differentiation, invasion, and motility in HeLa and SiHa human cervical cancer cell lines which express E6/E7 oncoproteins of HPV types 18 and 16, respectively. We found that SKI-606 significantly affects these processes in comparison with Iressa at the same concentration. These effects occur through the conversion of *β*-catenin's role from a transcription regulator to a cell-cell adhesion molecule via Src dephosphorylation.

## 2. Materials and Methods

### 2.1. Cell Cultures and Reagents

Human cervical cancer cell lines HeLa and SiHa were obtained from the American Type Tissue Culture, and maintained in DMEM (Life Technologies, Inc.) supplemented with 5% fetal bovine serum, 100 units/ml penicillin, 100 *μ*g/mL streptomycin, and 2 mM L-glutamine (Life Technologies, Inc.), and incubated at 37°C with 5% CO_2_ atmosphere. 

The dual Src and Abl tyrosine kinase inhibitor SKI-606 (Wyeth Research) and Iressa (AstraZeneca) were dissolved in DMSO (Sigma Chemical Co.), which was used as a solvent control in all experiments at the same volume used in the inhibitors; nevertheless, we did not observe any significant effect of DMSO-treated cells in comparison with untreated ones. Once cell cultures were 70% confluent, they were incubated with different concentrations of drugs as reported in the figure legends. All our experiments were performed in triplicates.

### 2.2. Proliferation Assay

HeLa and SiHa cells were seeded in flat-bottomed 96-well plates at 1 × 10^4^ cells per well in a volume of 100 *μ*l of supplemented medium. Three dilutions of SKI-606 and Iressa were added as indicated in the figure legends and the volume was adjusted to 200 *μ*l. Eight hours before harvesting onto glass fiber filters, 1 *μ*Ci [^3^H]thymidine was added to each well. Incorporation of [^3^H]thymidine was measured using a filter scintillation counter (1430 MicroBeta, Wallac). 

### 2.3. Cell Cycle Analysis

HeLa and SiHa cells were treated with 5 *μ*M SKI-606 and Iressa for two days. Cells were harvested, washed, and fixed and subsequently treated with 50 *μ*g/mL RNase and stained with 50 *μ*g/mL propidium iodide for 30 minutes. Cells were analyzed in a FACSCalibur and data were evaluated with Cell Quest and ModFitLT v3.1 software.

### 2.4. Cell Wounding Assay

HeLa and SiHa cervical cancer cells were grown to confluence and serum-starved for 24 hours, and finally wounded with a 2 mL pipette and treated with 5 *μ*M of SKI-606 and Iressa. Cells were examined by light microscopy 24 and 48 hours later for the ability to repopulate the wound as well as for cell migration.

### 2.5. Invasion Assay

Cell invasion was assayed in 24-well Biocoat Matrigel invasion chambers (8 *μ*m; Becton Dickinson) according to the manufacturer's protocol. Briefly, 5 × 10^4^ wild type and treated cells, with 5 *μ*M SKI-606 and Iressa, were plated in the top chamber. The bottom chamber contained DMEM medium. After 48 hours incubation, the noninvasive cells were removed with a cotton swab. The cells that migrated through the membrane and stuck to the lower surface of the membrane were fixed with methanol and stained with hematoxylin. For quantification, cells were counted under a microscope in five predetermined fields.

### 2.6. Soft Agar Growth Assay

5 × 10^3^ HeLa and SiHa cells (untreated and treated with 5 *μ*M SKI-606 and Iressa) were placed in DMEM medium containing 0.4% agar and plated over a layer of DMEM medium containing 0.7% agar. The cultures were examined every 1-2 days for 3 weeks.

### 2.7. Western Blot and Immunoprecipitation Analyses

Western blot was performed as previously described [21], for the experiments described in this study, anti-E-cadherin, *α*-catenin, *β*-catenin, *γ*-catenin, and P-cadherin monoclonal antibodies (mAbs) (Bio/Can Scientific); antifascin mAb (sc-21743, Santa Cruz Biotechnology Inc); anticyclin D1, cyclin D3, Cdk6, Id-1, IGF-R1 antisera (sc-753, sc-182, sc-177, sc-488 and sc-713, Santa, Cruz Biotechnology); anti-EGF-R antisera (Cell Signaling); anticyclin D2 mAb (Clone DCS-3, Sigma), anti-Cdk4 mAb (Clone DCS-35, Chemicon) and antiactin mAb (clone C4, Roche Diagnostics) were used for this assay. For immunoprecipitation, 300  *μ*g of proteins were immunoprecipitated with anti-*β*-catenin mAb (clone 14, Bio/Can Scientific). Immunoprecipitated samples were then blotted on a nitrocellulose membrane and detected with antiphosphotyrosine (clone 4G10, Upstate Biotechnology) or anti-*β*-catenin mAb.

### 2.8. Immunofluorescence Analysis

HeLa cells were seeded and treated with/without SKI-606 and Iressa, 5 *μ*M each, on cover-slips for 2 days at a density of 50,000-cells/35 mm dish. The cells were rinsed with PBS and fixed with 3% (w/v) paraformaldehyde in PBS for 5 minutes, followed by an incubation in precooled methanol (−20°C) for 15 minutes. The cells were then incubated with the primary antibody anti-E-cadherin, *α*-, *β*-, and *γ*-catenin (mAb) (Bio/Can Scientific) and with 0.2% BSA in PBS, as negative controls, for 1 hour at room temperature. After three washes with PBS, the cells were incubated with appropriate secondary antibody conjugated to FITC (Jackson Immunoresearch Laboratories) for 45 minutes. Finally, the cover-slips were washed with PBS, mounted with Airvol (Air Products and Chemicals, Inc.), and viewed with a Zeiss Axiophot fluorescent microscope equipped with 63X plan apochromat objectives and photographed.

## 3. Results

**Figure 1:**
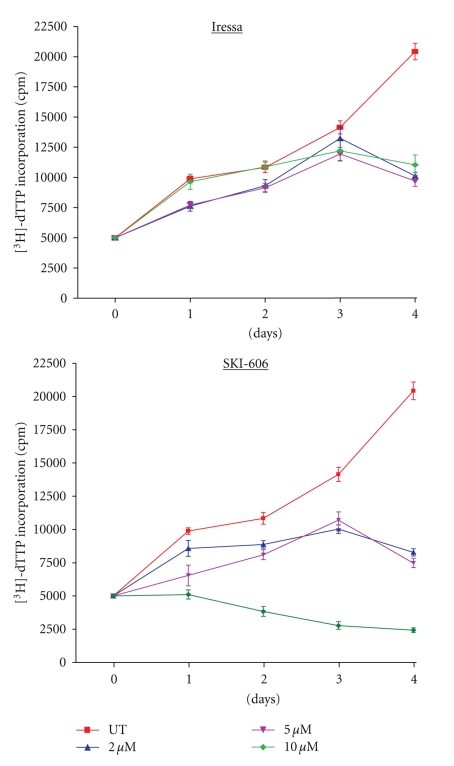
Effect of SKI-606 and Iressa on cell growth of HeLa human cervical cancer cells. We note that cell proliferation slightly relies on Src/Abl and EGF-R inhibitor concentrations at 2 and 5 *μ*M each; in contrast, Src/Abl inhibitor, but not EGF-R, drastically inhibits cell proliferation of HeLa and SiHa cells (data not shown) at 10 *μ*M.* Bars*, SDs of each point.

**Figure 2:**
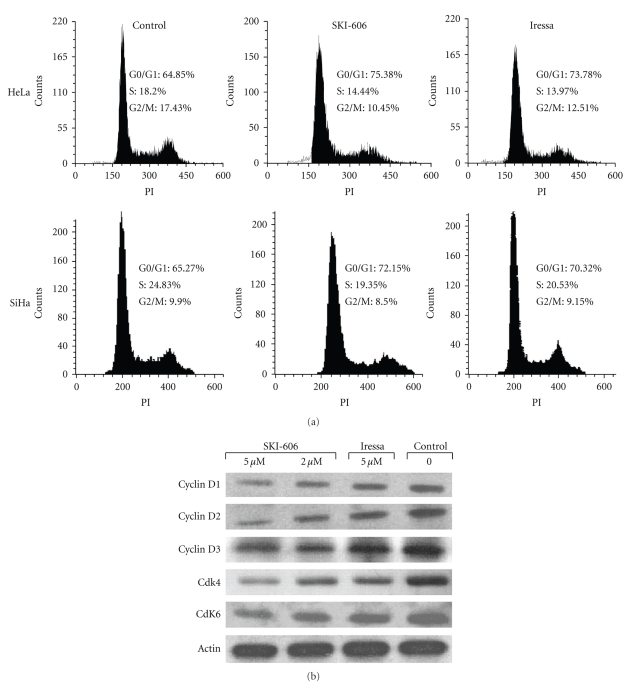
SKI-606 and Iressa (to a lesser extent) provoke G_0_-G_1_ cell cycle arrest and reduction of S and G_2_-M phase of HeLa and SiHa cells. *A*, propidium iodide staining shows a significant increase (*P* < .0001 and *P* < .001) in the proportion of HeLa and SiHa cells, respectively, in the G_0_-G_1_ phase of the cell cycle following 48 hours of SKI-606 and Iressa treatments (5 *μ*M). *B*, SKI-606, but not Iressa, treatment for 48 hours results in changes in key G_0_-G_1_ cell cycle phase regulators in HeLa cells.

SKI-606 is a Src/Abl kinase inhibitor that was shown to have antiproliferative effects on chronic myelogenous leukemia cells as well as breast, colon and nonsmall lung cancer cells [22–25]; however, the efficacy of SKI-606 in human cervical cancer is still unknown. Therefore, we compared the effect of SKI-606 with Iressa, a tyrosine kinase inhibitor (TKI) of EGF and ErbB2 receptors which is presently in phase II clinical trial for patients with recurrent or metastatic cervical carcinomas [26], in HeLa and SiHa cancer cell lines. Treatment of human cervical cancer cells for 1–4 days with 2 and 5 *μ*M of SKI-606 slightly increased the inhibition of cell proliferation when compared with cells treated with Iressa and untreated cells in both cell lines. Likewise, SKI-606, but not Iressa, caused a significant inhibition of cell proliferation of HeLa and SiHa cell lines at 10 *μ*M concentrations (Figure 1 and data not shown). To examine whether the antiproliferative effect of SKI-606 and Iressa on HeLa and SiHa cells was partly mediated via specific cell cycle arrest, we investigated cell cycle phase distributions of SKI 606 and Iressa-treated cells using flow cytometric analysis. Results revealed that SKI-606 and Iressa (to a lesser extent) cause accumulation of cells in the G_0_-G_1_ fraction. For instance, following exposure to 5 *μ*M SKI-606 and Iressa for 48 hours, the percentage of G_0_-G_1_ phase cells slightly increased in HeLa and SiHa cells, with a proportional reduction in S and G_2_-M phase fractions (Figure 2(a)). To investigate possible molecular mechanisms involved in SKI-606 and Iressa-mediated G_0_-G_1_ cell cycle arrest, several key molecules regulating transition from the G_1_ to the S cell cycle phase were examined in HeLa cells after 48 hours of SKI-606 and Iressa-treatment, including cyclin D1, D2 and D3 as well Cdk4 and 6. Results showed reductions in cyclin D1, D2, and D3 as well as Cdk4 and Cdk6 in HeLa-treated cells when compared with untreated cells (Figure 2(b)).

**Figure 3:**
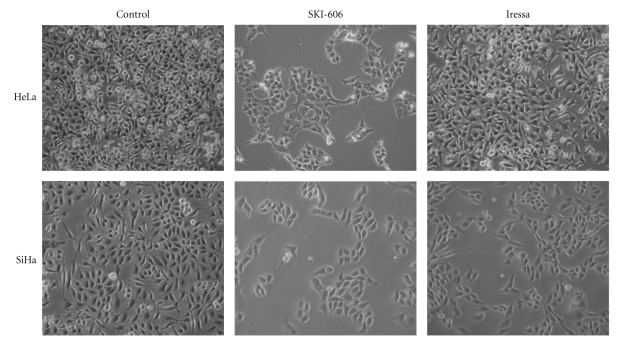
SKI-606 and Iressa (to a lesser extent) induce morphological changes in HeLa and SiHa cell lines. Untreated (control) cells possess a fibroblast-like (mesenchymal) cell phenotype, whereas 48 hours treatment with SKI-606 (5 *μ*M) significantly induces a mesenchymal-epithelial transition of these cells in comparison with Iressa-treated cells.

**Figure 4:**
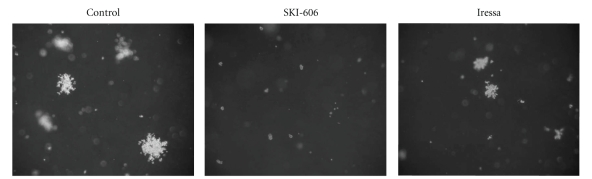
Effect of SKI-606 and Iressa on colony formation ability of HeLa cell line. HeLa cells are able to form large colonies in soft agar. In contrast, SKI-606 inhibits the colony formation ability of HeLa (*P* < .0001) and SiHa cells (data not shown) in soft agar, whereas, Iressa slightly reduce the colony formation following treatment with 5 *μ*M as described in the Materials and Methods section. Magnification is 100x. We have obtained the same results using SiHa cell line.

We also investigated the effect of SKI-606 and Iressa on cell differentiation in HeLa and SiHa cervical cancer cells. In the absence of treatment, HeLa and SiHa cells displayed a fibroblast-like (mesenchymal) morphology, and formed multilayered disorganized cells. In contrast, as indicated in Figure 3, blocking Src/Abl activity led to a considerable phenotypic conversion from fibroblast-like (mesenchymal) to epithelial phenotype. Cells became more flattened in appearance, and showed an increase in cell-cell contact in comparison with untreated cells and Iressa-treated cells in which these parameters are only slightly affected. Next, we examined the effect of SKI-606 and Iressa on colony formation of HeLa and SiHa cells; we found that SKI-606 significantly blocks colony growth, in soft agar, when compared with cells treated with Iressa and wild type cells (Figure 4).

**Figure 5:**
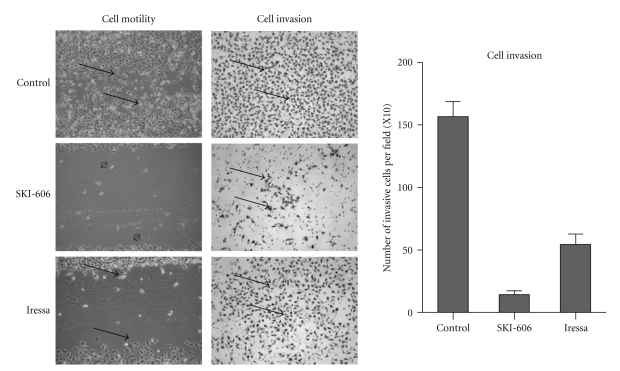
Comparison of SKI-606 and Iressa activity against cell migration and invasion ability of the HeLa cell line. SKI-606 and, to a lesser extent, Iressa block cell migration at the same concentrations, as indicated in Materials and Methods, (arrows) within 48 hours in comparison with untreated (control), (*P* < .001) using a cell wounding assay. In parallel, SKI-606 dramatically inhibits cell invasion ability of HeLa and SiHa cells (data not shown) in comparison with Iressa-treated and untreated (control) cells (arrows indicate invasive cells), (*P* < .0001) using Boyden chambers. Experiments conducted on SiHa cells showed the same data regarding cell migration and invasion ability.

**Figure 6:**
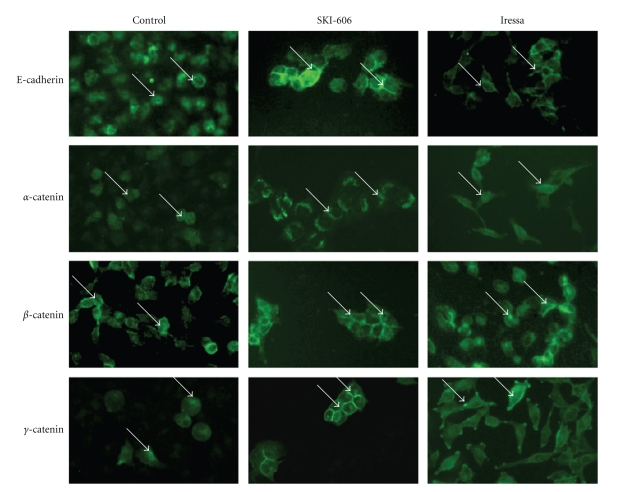
Immunofluorescence analysis of E-cadherin, *α*-, *β*-, and *γ*-catenin expression in HeLa-untreated (control) and SKI-606 as well as Iressa-treated cells. We note that SKI-606-treatment restores the expression patterns of E-cadherin and *α*-, *β*-, as well as *γ*-catenin, on cell surfaces and undercoat membrane, at 5 *μ*M concentration, in HeLa cells when compared with Iressa-treated and untreated cells at the same concentrations.

To evaluate the role of SKI-606 and Iressa on cell invasion and migration abilities of human cervical cancer cells, Matrigel invasion and wound-healing assays were preformed. HeLa and SiHa cells were treated for 24 and 48 hours with 5 *μ*M of SKI-606 and Iressa. As illustrated in Figure 5, SKI-606 blocked cell migration and invasion abilities of HeLa cells in comparison with Iressa-treated cells and wild type cells. We found that SKI-606 and Iressa affect cell migration and invasion of SiHa cells at the same level of HeLa cells. Next, we investigated the expression pattern of E-cadherin and *α*-, *β*-, and *γ*-catenin using Western blot and immunofluorescence methods in HeLa cells, untreated and treated with SKI-606 and Iressa. Using Western blot analysis, we found that SKI-606 slightly up-regulate the expression of the E-cadherin/catenin complex (data not shown). Immunofluorescence analysis results were consistently correlated with cell phenotype as well as invasion and migration; following SKI-606 treatment, typical cell-cell adhesion type staining for E-cadherin and *α*-, *β*-, as well as *γ*-catenin was observed on cell surfaces and the undercoat membrane in HeLa cells converted to an epithelial phenotype (Figure 6). In contrast, untreated cells and, to a lesser extent, those treated with Iressa showed a weak and diffused cytoplasmic and nuclear staining, with complete loss of membrane localization of E-cadherin and catenins (Figure 6).

**Figure 7:**
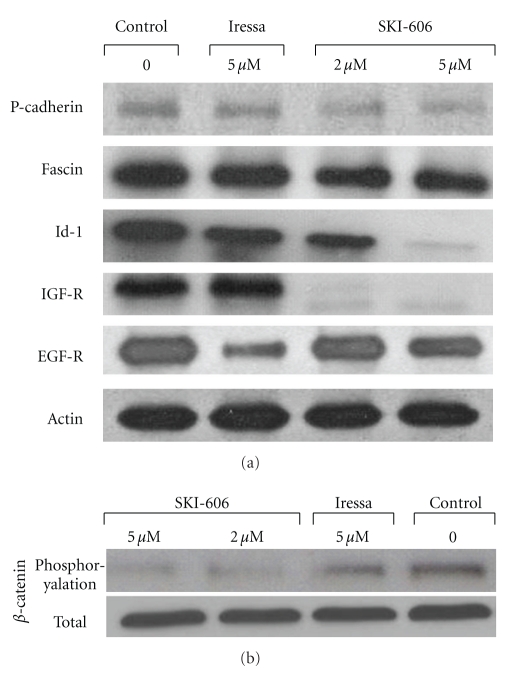
Comparison between the influence of SKI-606 and Iressa activity on P-cadherin, fascin, Id-1, IGF-R1, and EGF-R expression as well as *β*-catenin phosphorylation in HeLa cells. (a) Western blot analysis of P-cadherin, fascin, Id-1, IGF-R1, and EGF-R expression in HeLa-untreated and treated cells. We found that SKI-606 decreases the expression of P-cadherin, fascin, Id-1 and IGF-R1 in HeLa cells in comparison with Iressa-treated (as indicated in the Materials and Methods) and untreated cells; in contrast, Iressa largely reduces the expression of EGF-R in comparison with SKI-606. (b) Tyrosine phosphorylation analysis of *β*-catenin in HeLa cells and SKI-606 as well as Iressa-treated cells. Src/Abl inhibitor (SKI-606) blocks the constitutive phosphorylation of Src and consequently *β*-catenin phosphorylation in HeLa cancer cells. Cells were grown for two days in both the absence (control) and presence of 2 and 5 *μ*M of SKI-606 and 5 *μ*M of Iressa. Cell lysates were immunoprecipitated with anti-*β*-catenin antibody and analyzed by immunoblotting with antiphosphotyrosine antibody as described in Section 2.

To determine the role of Src/Abl inhibitor on the regulation pattern genes of cell invasion and migration, we examined the effect of SKI-606 on gene expression patterns of P-cadherin, fascin, Id-1, IGF-R1, and EGF-R, which are important initiators of cell invasion and motility in several human carcinomas including cervical, using Western blot analysis. We found that SKI-606 significantly decrease the expression of P-cadherin, fascin, Id-1, and IGF-R1 in HeLa cells in comparison with untreated cells and Iressa-treated cells which are only slightly affected (Figure 7(a)); moreover, SKI-606 and Iressa (to a larger extent) reduce the expression of EGF-R in comparison with control cells (Figure 7(a)). Regarding the mechanism of E-cadherin/catenin complex re-localization and P-cadherin, fascin, Id-1, IGF-R1, and EGF-R gene deregulations, we hypothesized that SKI-606 provokes E-cadherin/catenin complex reassociation by blocking the phosphorylation of *β*-catenin through the inhibition of pp60(c-Src) kinase phosphorylation in HeLa cancer cells. Using SKI-606, we recently demonstrated that *β*-catenin is physically associated with activated pp60(c-Src) kinase and constitutively phosphorylated in human colorectal and breast cancer cells [24, 27]. To assess this possibility in cervical cancer cells, we examined the effect of SKI-606 on *β*-catenin regulation patterns in HeLa cancer cells. The cells were treated for 48 hours with 5 *μ*M of SKI-606 and Iressa and then immunoprecipitated with anti-*β*-catenin. Western blot analysis with antiphosphotyrosine revealed that *β*-catenin is dephosphorylated in SKI-606-treated cells in comparison with untreated and Iressa-treated cells in which this parameter is only slightly affected (Figure 7(b)). In parallel, we found that SKI-606 inhibits *β*-catenin phosphorylation and consequently its translocation to the nucleus in these cells (Figure 6); thus, SKI-606 blocks cell migration through the conversion of *β*-catenin's role from a transcriptional regulator to a cell-cell adhesion function in HeLa-cervical cancer cells and by modulating the expression patterns of many genes including P-cadherin, fascin, Id-1, IGF-R1, and EGF-R (Figure 7(a)).

## 4. Discussion

In this study, we examined the effect of Src/Abl kinase inhibitor on two human cervical cancer cells. Src kinases are transducers of signals activated by many different classes of cell-surface receptors; more specifically, Src can be activated by growth factor receptors including ErbB and IGF-receptors, cytokine receptors, protein tyrosine phosphatase 1B, CAS, and focal adhesion kinase (FAK). In addition, Src interacts with a network of intracellular signaling pathways, including the integrin/FAK pathway, *β*-catenin/Wnt, RAS-MEK, phosphatidylinositol-3-OH kinase-AKT and Janus-activated kinase-STAT pathways [28–30]. These complex interactions explain why Src is involved in a large number of cellular functions. This has prompted the development of a number of small-molecule Src kinase inhibitors and tyrosine kinase inhibitor (TKI) of ErbB-receptors, such as SKI-606 and Iressa, which could be used to treat patients with metastatic cancers. Several studies have revealed that SKI-606 inhibits cell proliferation and blocks cell cycle progression, invasion and migration of several invasive human cancers including breast, colon and nonsmall lung cancer cells [23–25]. In parallel, numerous investigations found that Iressa decreases cell invasion and tumor formation of human cancer cells* in vitro* and *in vivo* [31–35]. In this paper, we report that Src/Abl and, to a lesser extent, EGF-R inhibitor decrease cell proliferation of two human cervical cancer cell lines, which is accompanied by a deregulation of cell cycle progression, particularly G_0_-G_1_ cell cycle. Therefore, these inhibitors down-regulate cyclin D1, D2, and D3 as well as their catalytic partners Cdk4 and Cdk6. We have recently found that D-type cyclins (D1, D2 and D3) as well as their catalytic partners Cdk4 and Cdk6 are downstream targets of cellular transformation induced by E6/E7 of HPV type 16 in mouse normal embryonic fibroblast cells ([21, 36, 37] and unpublished data). Herein, we demonstrate, for the first time, that SKI-606 and, to a lesser extent, Iressa block cell invasion and migration as well as colony formation in soft agar of HeLa and SiHa human cervical cancer cell lines which express E6/E7 oncoproteins of high-risk HPV types 18 and 16, respectively. In parallel, we reveal that SKI-606 and, to a lesser extent, Iressa, induces differentiation to an epithelial phenotype of HeLa and SiHa human cervical cancer cell lines; moreover, we report that Src/Abl inhibitor up-regulates and restores the expression patterns of E-cadherin as well as *α*-, *β*-, and *γ*-catenin in HeLa cells in comparison with untreated cells and Iressa-treated cells in which these parameters are less substantially affected. Other studies found that SKI-606 induces an over-expression of E-cadherin in human breast cancer cells [23]. Recently, Vultur et al. [38] demonstrated that Src/Abl inhibitor reduces cell invasion and migration abilities of primary human breast cancer cells by increasing membrane-localization of E-cadherin and *β*-catenin.

It is well established that high-risk HPV infection plays an important role in the progression of human cervical cancer. Moreover, the presence of high-risk HPVs has been shown to serve as prognostic factors in early-stage cervical cancer, and is associated with vascular invasion, lymph node metastases, and tumor size [7, 39, 40]. Nevertheless, during high-risk HPV infection, E6/E7 oncoproteins are expressed and, as a result, the restraint on cell-cycle progression is abolished and normal terminal differentiation is retarded [41]. Therefore, E6/E7 of high-risk HPV can deregulate several oncogenes, such as P-cadherin, fascin, Id-1, IGF-R1, and EGF-R which are known to enhance the progression of human cervical cancer [42–46]. In the present study, we provide evidence that SKI-606 and, to a lesser extent, Iressa inhibit cell invasion and migration of HeLa and SiHa cancer cells; this is accompanied by a downexpression of P-cadherin, fascin, Id-1, IGF-R1 and EGF-R. Consequently, the inhibition of cell invasion and migration by this Src inhibitor is related to the downregulation of those genes and probably other oncogenes that might be involved in this process through *β*-catenin's role conversion in human cervical cancer cells expressing E6/E7 oncoproteins of high-risk HPV. We have recently reported that *β*-catenin is physically associated and activated by pp60(c-Src) kinase and is constitutively phosphorylated on the tyrosine residue in human colorectal and breast cancer cells [24, 27]; therefore, Src activation converts *β*-catenin's role from a cell-cell adhesion molecule to a transcriptional regulator via its interaction with the Tcf/Lef family of transcription factors [47, 48]. Our present data show that Src/Abl inhibitor restores the expression of *β*-catenin to the undercoat membrane to act as a cell-cell molecule which can deregulate several genes including P-cadherin, fascin, Id-1, IGF-R1 and EGF-R; thus, these deregulations induce cell differentiation and consequently block cell invasion ability of these human cervical cancer cells.

In conclusion, our study is the first evidence demonstrating that treatment with Src/Abl inhibitor and, to a lesser extent, EGF-R inhibitor induce differentiation to an epithelial phenotype and upregulates as well as restores the expression of the E-cadherin/catenin complex and subsequently inhibits cell invasion and migration of human cervical cancer cells. These observations are accompanied by downregulation of several important regulators of cell invasion and metastasis. Thus, this investigation has several clinical implications. First and most importantly, it points toward a new mechanism of action for Src inhibitors and suggests the use of P-cadherin, fascin, Id-1, IGF-R1 and EGF-R as biomarkers. Second, we speculate that therapeutic strategies based on Src inhibitor can dramatically reduce human tumor recurrence and metastasis. Beyond this, based on the implication of Src and *β*-catenin in human carcinogenesis, the findings described in the present report may lead to advances in the biology and treatment of malignant cervical carcinomas which are positive for high risk HPVs.
